# Antiviral activity of silymarin in comparison with baicalein against EV-A71

**DOI:** 10.1186/s12906-020-2880-2

**Published:** 2020-03-23

**Authors:** Salima S. Lalani, Mohd Ishtiaq Anasir, Chit Laa Poh

**Affiliations:** Centre for Virus and Vaccine Research, Sunway University, Bandar Sunway, 47500 Subang Jaya, Selangor Malaysia

**Keywords:** Hand, Foot and mouth disease (HFMD), Enterovirus-71 (EV-A71), Silymarin, Baicalein, Flavonoids, Virucidal, Antivirals, Attachment inhibitor

## Abstract

**Background:**

The hand, foot and mouth disease (HFMD) is a febrile and exanthematous childhood disease mainly caused by Enterovirus 71 (EV-A71). In severe HFMD, virulent EV-A71 strains can cause acute flaccid paralysis and cardiopulmonary edema leading to death. Currently, no FDA approved antiviral treatment or vaccine is available for EV-A71. Flavonoids such as silymarin and baicalein are known to possess in vitro antiviral properties against viruses. In this study, the cytotoxicity and antiviral activity of silymarin, baicalein and baicalin were investigated.

**Methods:**

The cytotoxic effects of three flavonoids towards rhabdomyosarcoma (RD) cells were first examined using cell proliferation MTS [3-(4,5-dimethylthiazol-2-yl)-5-(3-carboxymethoxyphenyl)-2-(4-sulfophenyl)-2H-tetrazolium] assay. Compounds found to be non-cytotoxic in RD cells were evaluated for their in vitro antiviral properties against the EV-A71 subgenotype B4 strain 41 (5865/SIN/000009) using antiviral assays. Viral infectivity was determined by reduction of the formation of plaques in RD cells. For the measurement of RNA copy number, the real time quantitative reverse transcription PCR (qRT-PCR) was used. The most potent compound was further evaluated to determine the mode of action of inhibition by time course, virus attachment and entry assays in Vero cells.

**Results:**

Silymarin was shown to exert direct extracellular virucidal effects against EV-A71 at 50% inhibitory concentration (IC_50_) of 15.2 ± 3.53 μg/mL with SI of 10.53. Similarly, baicalein exhibited direct extracellular virucidal effects against EV-A71 at a higher IC_50_ value of 30.88 ± 5.50 μg/mL with SI of 13.64. Besides virucidal activity, silymarin was shown to block both viral attachment and entry of EV-A71 to inhibit infection in Vero cells.

**Conclusions:**

Silymarin has a stronger inhibition activity against EV-A71 in comparison to baicalein. It could serve as a promising antiviral drug to treat EV-A71 infections.

## Background

The hand, foot and mouth disease (HFMD) is a febrile and exanthematous disease characterized by painful ulcerative lesions in the mouth and blisters on the hands, feet, groin, and buttocks. Enterovirus 71 (EV-A71) and Coxsackievirus 16 (CV-A16) are the main etiological agents of HFMD [1]. Virulent strains of EV-A71 can cause severe HFMD associated with acute flaccid paralysis and cardiopulmonary edema which could lead to death. An epidemiological study of HFMD in China from 2008 to 2017 reported that there were more than 18 million HFMD cases including 153,436 severe and 3633 fatal cases during this period. EV-A71 was the predominant enterovirus serotype of severe and fatal cases, constituting 70.03 and 92.23%, respectively [2]. Thus, HFMD caused by EV-A71 is considered as a global health threat, especially for children under the age of 5 years [3].

With the rising concern of HFMD outbreaks caused by EV-A71, there is an urgent need for medical interventions to treat or prevent EV-A71 infection. Currently, no FDA approved antiviral treatment against EV-A71 is available. A vaccine against EV-A71 has been approved in China but it is still under surveillance after Phase III clinical trials [4]. Management of severe cases of HFMD caused by EV-A71 infection primarily focused on palliative care such as the use of intravenous fluid therapy to prevent dehydration. In addition, milrinone therapy was introduced to treat EV-A71-induced pulmonary edema [5].

Many plants are known to synthesize natural products with medicinal properties. Arrays of biologically active compounds found in plants have been shown to be effective against diverse viruses. For instance, raoulic acid, ursolic acid, matrine, chrysosplenetin, and penduletin have all been shown to inhibit EV-A71 replication in vitro [6]. In addition, a group of polyphenolic compounds from plants known as flavonoids have been shown to exhibit antiviral properties against various viruses [7, 8]. Recent flavonoid compound library screening has identified quercetagetin and peracetate pulicarine to be potent inhibitors of EV-A71 in vitro [9].

Silymarin (extracted from milk thistle) was shown to sequester the replicase system of the chikungunya virus (CHIKV) [10]. Silymarin was also identified to limit various steps of the hepatitis C virus (HCV) replication and translation [11]. In addition, silymarin suppressed the TNF-α activation of NF-κB dependent transcription by inhibiting NS5B polymerase in HCV infection [12]. Previously, silymarin was found to be safe in adults and children during a clinical trial to evaluate its hepatoprotective properties in patients with liver cirrhosis [13]. Thus, silymarin is a promising antiviral candidate to be further developed based on its safety and therapeutic potential.

Baicalein and its metabolite baicalin (found in plant *Scutellaria baicalensis*) were previously shown to exhibit anti-inflammatory, antioxidant and free radical scavenging effects [14, 15]. Recently, baicalin was reported to inhibit replication of dengue virus by interfering with viral attachment to Vero cells. The exact antiviral mechanism of baicalin against dengue infection is still unknown [7]. Another study by Li et al. (2015) reported that baicalin exhibited antiviral activity against EV-71 strain BrCr-TR by suppressing the Fas/FasL pathway and 3D polymerase [16]. Similar to baicalin, its parent compound baicalein was shown to inhibit dengue virus in vitro [17]. Other than dengue virus, baicalein was reported to inhibit the H5N1 neuraminidase and HIV reverse transcriptase [18, 19].

In the absence of a vaccine and therapeutic agent against EV-A71, the search for an effective antiviral is indispensable. Therefore, in this study, we aimed to evaluate antiviral potentials of silymarin and baicalein against EV-A71 in vitro. We examined the antiviral effects of silymarin, baicalein and baicalin against EV-A71 [sub-genotype B4 strain 41(5865/SIN/000009, GeneBank accession number AF 316321)] infections in Rhabdomyosarcoma (RD) and Vero cells. Baicalin was included in this study to serve as a positive control as it was previously shown to exert antiviral effects against EV-A71 [16]. Out of the three flavonoids under investigation, only silymarin and baicalein have the potential to inhibit EV-A71, specifically by direct extracellular virucidal action. In addition, we showed that silymarin interfered with EV-A71 attachment and entry to Vero cells. Intriguingly, this is the first report indicating direct extracellular virucidal effects of silymarin and baicalein against EV-A71.

## Methods

### Virus and cell culture

Enterovirus (EV)-A71 strain 41 (5865/SIN/000009) was propagated in rhabdomyosarcoma (RD, ATCC# CCL-136) cells with Dulbecco’s Modified Eagle’s Medium (DMEM) supplemented with fetal bovine serum (FBS) and 1% penicillin-streptomycin antibiotics (PSA). Briefly, a monolayer of confluent RD cells in a T-75 flask was infected with EV-A71 virus and adsorption was allowed for 1 h at 37 °C in an incubator supplemented with 5% CO_2_. After incubation, the inoculum was removed and maintenance media (DMEM supplemented with 2% FBS) was added. Cytopathic effect (CPE) of RD cells was monitored over a period of 24–48 h and the virus was harvested by three freeze-thaw cycles. The virus was aliquoted and stored at -80 °C until further use. Vero (ATCC® CCL-81) cells were grown in DMEM supplemented with 10% FBS and 1% PSA.

### Flavonoid compounds

Silymarin, baicalein, and baicalin were purchased from Sigma-Aldrich (St. Louis, MO, USA). Each of the flavonoid compounds was dissolved in dimethyl sulfoxide (DMSO) (St. Louis, MO, USA) to yield 50 mg/mL stocks. The stocks were aliquoted and kept at − 80 °C until use. Required concentrations of each flavonoid were made by dilutions with serum-free DMEM media or DMEM supplemented with 2% FBS and 1% PSA. The diluted solutions were filtered with a 0.2 μm pore size filter (Millipore, MA, USA) and kept on ice until use.

### Cytotoxicity assay

CellTiter 96® AQueous One Solution Cell Proliferation MTS [3-(4,5-dimethylthiazol-2-yl)-5-(3-carboxymethoxyphenyl)-2-(4-sulfophenyl)-2H-tetrazolium] assay kit (Promega, Madison, WI, USA) was used to evaluate the cytotoxicity of the three flavonoids according to the manufacturer’s instruction. Briefly, RD or Vero cells were seeded overnight in 96 well plates with a density of 2 × 10^5^ cells/mL. Cells were then treated with various concentrations of silymarin (6.25, 12.5, 25, 50,100 and 200 μg/mL), baicalein (1.68, 3.37, 6.75, 13.5, 27, 54 and 108, μg/mL) and baicalin (2.78, 5.57, 11.15, 22.31, 44.63 and 89.26 μg/mL) prepared in maintenance media. RD or Vero cells without test compounds served as a negative control. The plate was further incubated for 24 h at 37 °C. An aliquot of 20 μL of MTS solution was added to each well and the plate was incubated for 1 h at 37 °C. The absorbance was measured at 490 nm with an Infinite 200 Pro Multiplate Reader (Tecan, Männdedorf, Switzerland) (Supplementary Figure S1). The half-maximal cytotoxic concentration (CC_50_) and 10% cytotoxic concentration (CC_10_) for each flavonoid were determined using GraphPad Prism software, version 7 (GraphPad Software, Calif, USA).

### Plaque assay

The plaque assay was performed using the method described by Tan et al. (2012) with minor modifications [20]. Briefly, 6 well plates with 6 × 10^5^ RD cells/mL were prepared and incubated to grow overnight at 37 °C. The supernatants collected from antiviral assays were serially diluted (10-folds) and the cell monolayer was infected with each dilution. The plates were incubated at 37 °C for 1 h. After removal of the inoculum, 1.5–2 mL of 0.9% of high viscosity carboxy methyl cellulose (CMC) (Sigma Aldrich, MO, USA) with 2% FBS in DMEM (overlay media) were added to each well. After incubation for 3 days, the overlaid media was removed. The cells were washed three times with phosphate buffered saline (PBS), fixed with 3.7% formaldehyde and stained with 0.5% crystal violet. The plaques were counted manually against a white background and pictures were taken using the CTL Immunospot® S6 Versa (Cleveland, OH, USA). All experiments were performed as three independent replicates.

### Real-time, quantitative reverse transcriptase polymerase chain reaction (qRT-PCR)

The primers and probes used for the VP1 mRNA quantification by qRT-PCR were previously described by Tan et al. (2012) [20]. The forward primer 5′-GAGCTCTATAGGAG-ATAGTGTGAGTAGGG-3′, the reverse primer 5′-ATGACTGCTCACCTGCGTGTT-3′, and the TaqMan probe 5′6-FAM-ACTTACCCA/ZEN/ GGCCCTGCCAGCTCC-Iowa Black FQ-3′ were used in this study.

RNA extractions were performed using the QIAamp ViralRNA mini kit (Qiagen, Hilden, Germany) according to the manufacturer’s instructions. TaqMan® Fast virus 1-step master mix (ABI, Carlsbad, Calif, USA) was used to carry out qRT-PCR in the CFX96 TouchTM Real-Time PCR Detection System (Bio-Rad, Calif, USA). The cDNA synthesis was carried out by reverse transcription for 5 min at 50 °C and subsequently amplified for 40 cycles at 95 °C for 3 s, 60 °C for 30 s. A standard graph was plotted using serial dilutions of the standard viral stock. The threshold cycle value (Cq) was calculated using the default setting of the machine. All experiments were performed in triplicates.

### Antiviral assays

#### Virucidal assay

RD cells (2 × 10^4^/well) were grown overnight in wells of a 96 well plate. The virus was pre-treated with each of the flavonoids for 1 h at MOI of 1 (pfu/ml = 2 × 10^4^/0.1 mL). Cells were infected with the pre-treated virus-flavonoid-mixture. The inoculum was allowed to incubate with the RD cells at 37 ° for 1 h. After 1 h, the inoculum was removed, cells were washed with PBS and maintenance media was replaced_._ After 24 h, the supernatant was collected and infectious viral titers were quantified using the plaque assay [20]. RNA copy number was determined by qRT-PCR [21] [Supplementary Figure S2(a)].

#### Time course assay for virucidal activity

RD cells (2 × 10^4^/well) were grown overnight in wells of a 96 well plate. The virus was pre-treated with 100 μg/mL silymarin or 54 μg/mL of baicalein for 15, 30, 45 and 60 min at MOI of 1 (pfu/ml = 2 × 10^4^/0.1 mL) for 1 h. Cells were infected with the pre-treated virus-flavonoid-mixture and allowed to incubate with the RD cells at 37 °C for 1 h. After 1 h, the inoculum was removed, cells were washed with PBS and maintenance media was added. After 24 h, the supernatant was collected and infectious viral titers were quantified using the plaque assay [20]. To determine the early time points for the virucidal activity of silymarin, the flavonoid treated-virus mixture was assayed at 1, 5, 10, 15, 20, 40 and 50 min [Supplementary Figure S2(b)].

#### Cell protection assay

RD cells (2 × 10^4^/well) were seeded overnight in each well within a 96 well plate. Cells were treated with different concentrations of a flavonoid for 1 h at 37 °C. After incubation, flavonoid containing media was removed and cells were washed with PBS. Pre-treated cells were infected with EV-A71 at MOI of 1 (pfu/ml = 2 × 10^4^/0.1 mL) for 1 h. The inoculum was removed, cells were washed with PBS and replaced with 2% FBS supplemented DMEM. The supernatant was collected after 24 h and the infectious viral titers were quantified using the plaque assay [20]. RNA copy number was determined using qRT-PCR [21] [Supplementary Figure S2(c)].

#### Post-infection assay

RD cells (2 × 10^4^/well) were grown overnight in wells of a 96 well plate. The cells were infected with the virus at MOI of 1 (pfu/ml = 2 × 10^4^/0.1 mL) for 1 h and incubated at 37 °C. The inoculum was removed and RD cells were washed with PBS. The virus-infected cells were treated with serially diluted concentrations of each flavonoid prepared in maintenance media and incubated for 24 h at 37 °C. After 24 h, the supernatant was collected and infectious viral titers were quantified using the plaque assay [20]. RNA copy number was determined using qRT-PCR [21] [Supplementary Figure S2(d)].

#### Comprehensive assay

RD cells (2 × 10^4^/well) were grown overnight in wells of a 96 well plate. A monolayer of RD cells was pre-treated with various concentrations of a flavonoid for 1 h and incubated at 37 °C. Simultaneously, the virus at MOI of 1 (pfu/ml = 2 × 10^4^/0.1 mL) was pre-treated with the same concentrations of the flavonoid for 1 h at 37 °C. After incubation, flavonoid containing media was removed and the RD cells were washed with PBS. The flavonoid-treated virus was added to the pre-treated RD cells for 1 h at 37 °C. The inoculum was removed, RD cells were washed with PBS and replaced with the maintenance media (DMEM supplemented with 2% FBS). After 24 h, the supernatant was collected and infectious viral titers were quantified using the plaque assay [20]. RNA copy number was determined using qRT-PCR [21] [Supplementary Figure S2(e)].

#### Attachment assay

The attachment assay was performed using the method previously described by Yen et al. (2018) [22]. Briefly, Vero cells (1.5 × 10^5^/mL) were grown overnight in 6 well plates. Silymarin (100 μg/mL) was pre-incubated with EV-A71 (MOI = 1) at 37 °C for 1 h. Pre-chilled cells were infected with the pre-chilled silymarin-treated virus and incubated at 4 °C for 1 h to allow virus attachment. The inoculum was removed after 1 h and Vero cells were washed with PBS. CMC (1.2%, medium viscosity) overlay maintenance media was added to each well. After incubation for 3 days, the overlay media was removed. The Vero cells were washed three times with PBS, fixed with formaldehyde and stained with 0.5% crystal violet. The plaques were counted manually against a white background and pictures were taken using CTL Immunospot® S6 Versa [Supplementary Figure S3(a)].

#### Entry assay

To assess the effect of silymarin on the entry of EV-A71 into the Vero cells, an assay was performed using the method previously described by Yen et al. (2018) [22]. Briefly, the virus in the absence of silymarin was added to the pre-chilled Vero cells and incubated at 4 °C for 1 h to allow virus attachment. The inoculum was removed after 1 h and Vero cells were washed with PBS to remove any unattached virus. Silymarin (100 μg/mL) was added to Vero cells and the temperature was shifted to 37 °C for 1 h to allow virus entry. After 1 h, the medium was removed and Vero cells were treated with alkaline PBS (pH 11) for 60 s at room temperature to inactivate the extracellular virus. After 60 s, the alkaline pH was neutralized by the addition of PBS (pH 3) in each well. Cells were then washed with serum-free media. CMC (1.2%, medium viscosity) overlay maintenance media was added to each well. After incubation for 3 days, the overlay media was removed. The Vero cells were washed three times with PBS, fixed with formaldehyde and stained with crystal violet. The plaques were counted manually against a white background and pictures were taken using CTL Immunospot® S6 Versa [Supplementary Figure S3(b)].

### Data analysis

Data were analyzed using GraphPad Prism, version 7 (GraphPad Software, Calif, USA) to determine the 10% inhibitory concentration (CC_10_), half maximal cytotoxic concentration (CC_50_) and 50% inhibitory concentration (IC_50_). Data presented are the average ± S.E.M of three independent experiments and analyzed by student *t*-test (*p < 0.05, **p < 0.01, *** *p* < 0.001).

## Results

### Cytotoxicity effects of silymarin, baicalein and baicalin in RD cells

**Fig. 1:**
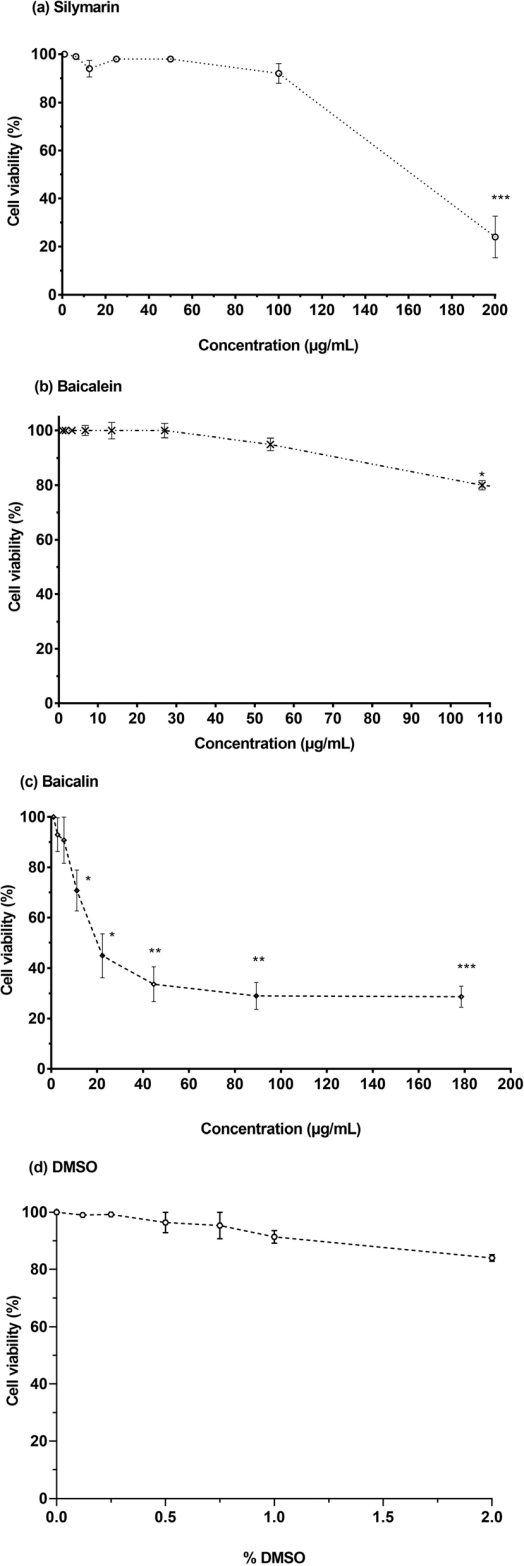
Cytotoxic effects of (**a**) silymarin, (**b**) baicalein (**c**) baicalin and (**d**) DMSO in RD cells. Flavonoids were diluted serially in DMEM containing 2% FBS and Rhabdomyosarcoma (RD) cells were treated with the diluted flavonoids under investigation for 24 h. Cytotoxicity was determined by the MTS assay using microplate reader to measure absorbance at 490 nm. Data are presented as mean ± S.E.M. Error bars indicate the range of values obtained from three independent experiments *p < 0.05, **p < 0.01, ***p < 0.001 indicates a significant difference compared to the negative control analysed by *t*-test

The cytotoxicity of silymarin, baicalein and baicalin in the RD cells was evaluated over a wide dose range using the MTS assay (Table 1). DMSO was used to dissolve the flavonoids and was found to be non-toxic up to 0.8% (Fig. 1d). No significant cytotoxic effects in RD cells were observed for silymarin and baicalein when these compounds were incubated for 24 h with RD cells at a concentration of up to 100 μg/mL and 54 μg/mL, respectively (Fig. 1a and b). The half maximal cytotoxic concentration (CC_50_) of silymarin was 160.20 μg/mL while the CC_50_ of baicalein was 421.46 μg/mL. The 10% cytotoxic doses (CC_10_) for silymarin and baicalein were 94.63 μg/mL and 90.93 μg/mL, respectively. This indicated that the viability of flavonoid-treated RD cells was more than 90% in comparison with the untreated cells (negative control) at these concentrations. Hence, 100 μg/mL of silymarin and 54 μg/mL of baicalein were chosen as the highest test concentrations for antiviral assays. In contrast, baicalin was poorly tolerated by the RD cells (Fig. 1c). Based on the CC_50_ and CC_10_ values, baicalin appeared to be significantly more toxic than silymarin and baicalein (Table 1). The CC_50_ and CC_10_ of baicalin were 42.18 μg/mL and 5.46 μg/mL, respectively. Due to its cytotoxicity, the antiviral property of baicalin was evaluated at its non-cytotoxic doses of 2.78 μg/mL and 5.57 μg/mL.

**Table 1:** Cytotoxicity of flavonoids against RD cells

Compound	CC_**50**_	CC_10_
Silymarin	160.20 ± 1.56 μg/mL	94.63 ± 2.29 μg/mL
Baicalein^a^	421.46 ± 3.23 μg/mL	90.93 ± 3.94 μg/mL
Baicalin	42.21 ± 4.15 μg/mL	5.46 ± 5.02 μg/mL

Different concentrations of silymarin, baicalein and baicalin were tested for cytotoxicity in RD cells by the MTS assay. CC_50_ refers to half maximal cytotoxic concentration whereas CC_10_ is the toxic dose at 10%. Data are presented as mean ± standard error of the mean (S.E.M)

^a^CC_50_ for baicalein is an extrapolated value as the value obtained is above the tested concentration of 108 μg/mL

### Silymarin and baicalein exhibited extracellular virucidal effects against EV-A71

**Fig. 2:**
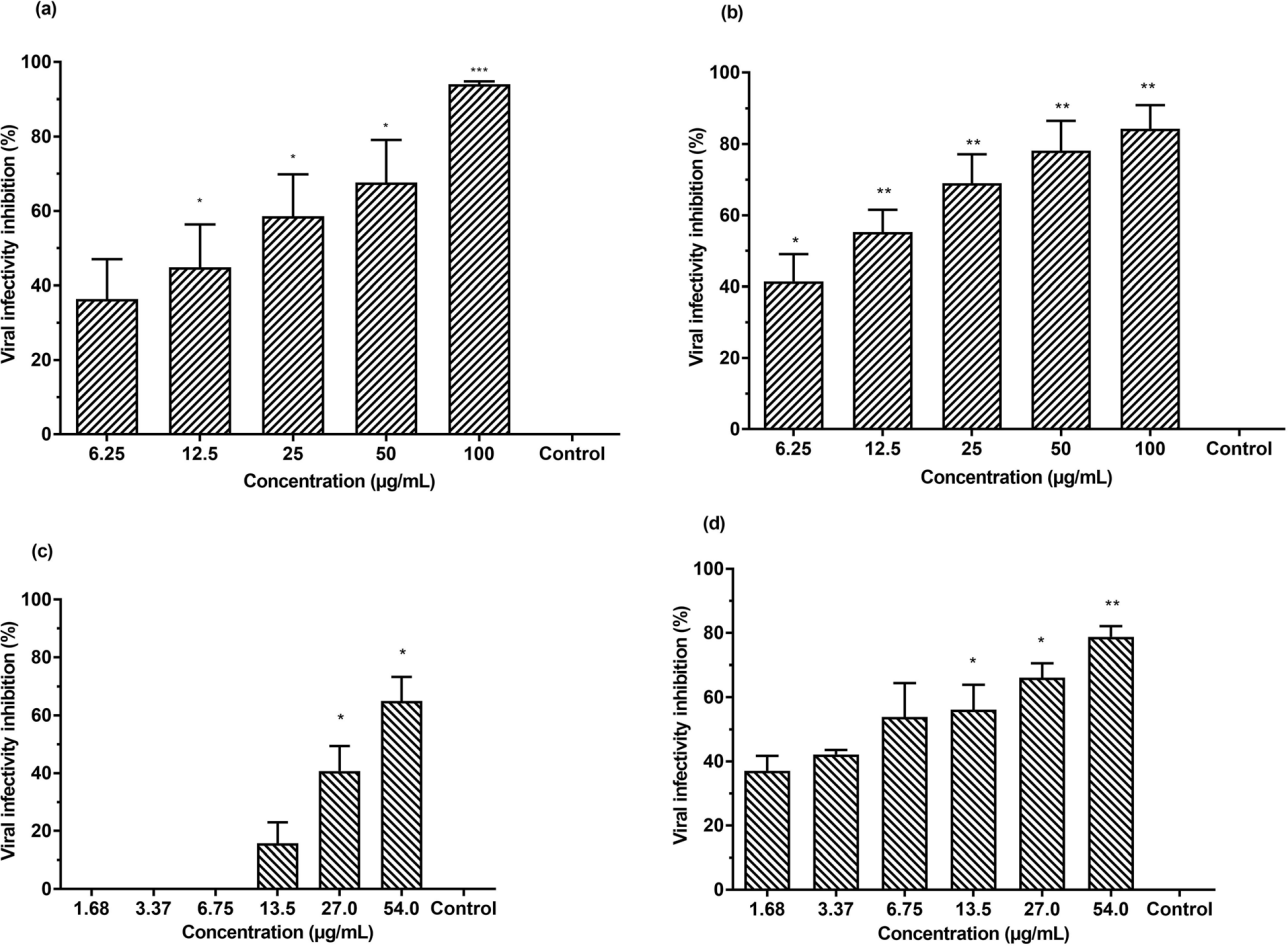
Silymarin and baicalein exhibited extracellular virucidal effects against EV-A71 in a dose-dependent manner. Inhibition of viral infectivity by silymarin determined by 2(**a**) plaque assays and 2(**b**) quantification of RNA copy number. Inhibition of viral infectivity by baicalein determined by 2(**c**) plaque assays and 2(**d**) quantification of RNA copy number. Data presented as mean ± S.E.M. Error bars indicate the range of values obtained from three independent experiments. *p < 0.05, **p < 0.01, ***p < 0.001 indicates a significant difference compared to the negative control analyzed by *t*-test

Virucidal assays were performed to investigate direct extracellular virucidal effects of silymarin and baicalein on EV-A71. Virucidal assays indicated both silymarin and baicalein exerted direct extracellular virucidal effects on EV-A71 in a dose-dependent manner. The half maximal inhibitory concentration (IC_50_) values of silymarin and baicalein determined by plaque assays were 15.20 ± 3.53 μg/mL and 30.88 ± 5.50 μg/mL, respectively. The highest inhibition of viral infectivity of EV-A71 by silymarin was 94.0% when the virus was treated with 100 μg/mL silymarin (Fig. 2a) whereas the highest infectivity inhibition of EV-A71 by baicalein was 65.0% when the virus was treated with 54 μg/mL baicalein (Fig. 2c). Consistently, RNA copy number quantifications indicated that the infectious viral titers of EV-A71 were reduced when EV-A71 were treated with either silymarin or baicalein prior to its infection of RD cells. Silymarin at 100 μg/mL was able to inhibit 84.3% whilst baicalein at 54 μg/mL inhibited 78.8% of EV-A71 viral infectivity based on RNA copy number quantifications (Fig. 2b and d). The selectivity index (SI) values were calculated as the CC_50_ value over the IC_50_ value for each compound. The SI values for silymarin and baicalein from virucidal assays are 10.53 and 13.64, respectively (Table 2). The SI values of both flavonoids indicated that these compounds are safe and efficacious as antivirals. In contrast to silymarin and baicalein, baicalin did not show any antiviral activity against EV-A71 at the non-cytotoxic doses (2.78 μg/mL and 5.57 μg/mL).

**Table 2:** Half-maximal inhibitory concentration (IC_50_) and selective index (SI) of silymarin and baicalein

Compound	Quantification	Comprehensive Assay		Virucidal Assay	
		IC_**50**_ (μg/mL)	SI	IC_**50**_ (μg/mL)	SI
Silymarin	Plaque assay	7.99 ± 3.0	20.05	15.2 ± 3.53	10.53
Baicalein	Plaque assay	2.27 ± 4.93	185.6	30.88 ± 5.50	13.64

IC_50_ refers to 50% inhibitory concentration required to inhibit the virus. SI is a selectivity index (CC_50_/IC_50_)

### Silymarin required a short period of contact with EV-A71 to exert its virucidal effects

**Fig. 3:**
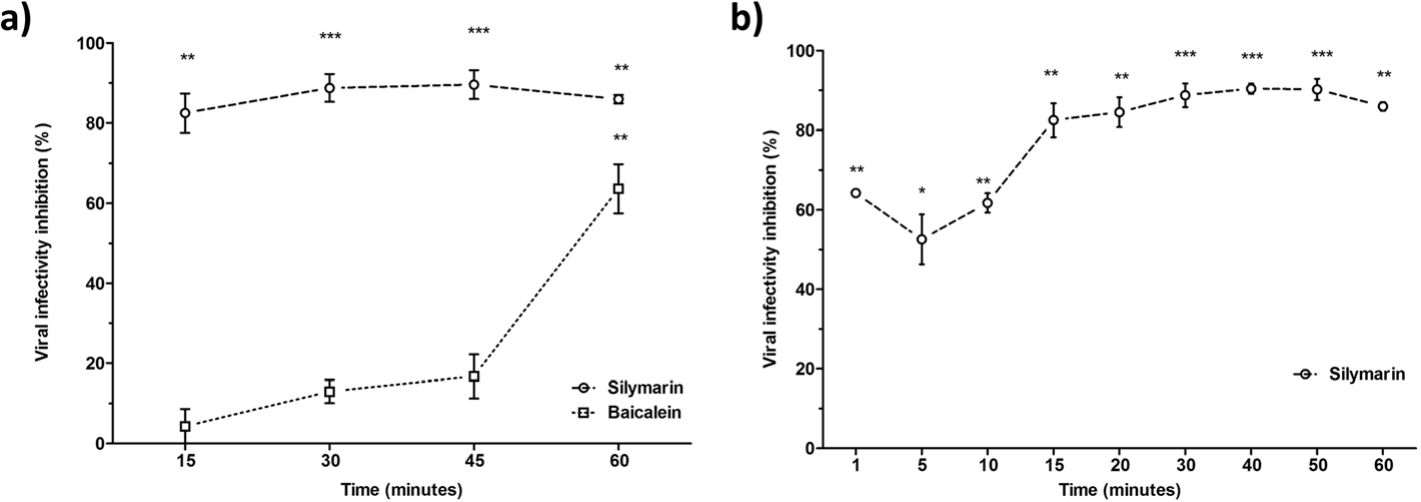
Extracellular virucidal activities of silymarin and baicalein against EV-A71 at different co-incubation times determined by plaque assays. **a** Silymarin (100 μg/mL) or baicalein (54 μg/mL) was co-incubated with EV-A71 for 15, 30, 45 and 60 min at 37 °C. The mixtures from each time point were subsequently inoculated into RD cells. **b** Silymarin (100 μg/mL) was co-incubated with EV-A71 for 1, 5, 10, 15, 20, 30, 40, 50 and 60 min at 37 °C. The mixtures from each time point were subsequently inoculated into RD cells. Data presented as mean ± S.E.M. Error bars indicate the range of values obtained from three independent experiments. *p < 0.05, **p < 0.01, ***p < 0.001 indicates a significant difference compared to the negative control (0 μg/mL) analysed by *t*-test

To determine the effective time needed for silymarin and baicalein to exert its virucidal effects on EV-A71, either of the flavonoids was co-incubated with EV-A71 at 37 °C for 15, 30, 45 and 60 min. Silymarin required a minimal co-incubation time of 15 min to elicit more than 80% viral infectivity inhibition. In contrast, baicalein required a longer period of 60 min of co-incubation time to exert 60% virucidal effects against EV-A71 (Fig. 3a).

To investigate the shortest time required by silymarin to exert its extracellular virucidal effects on EV-A71, the experiment was repeated using a different set of co-incubation times (1, 5, 10, 20, 40, 50 and 60 min) with silymarin against EV-A71. Silymarin required minimal co-incubation time from as low as 1 min to start exerting 65% virucidal effects on EV-A71, whilst maximum inhibition of 85% was achieved when silymarin was co-incubated with EV-A71 for 30 min (Fig. 3b).

### Silymarin and baicalein exhibited minimal cell protection effects on RD cells against EV-A71

**Fig. 4:**
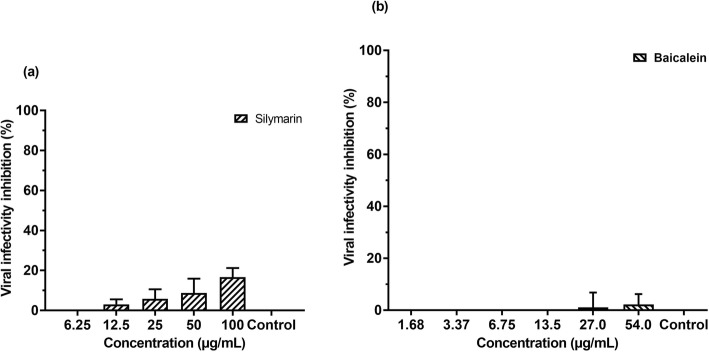
Effects of silymarin on cell protection against EV-A71 determined by plaque assays. **a** Inhibition of viral infectivity by silymarin 4 (**b**) Inhibition of viral infectivity by baicalein. Data presented as mean ± S.E.M. Error bars indicate the range of values obtained from three independent experiments

In order to determine the cell protection effects of silymarin and baicalein in RD cells, the cell protection assays were performed by incubating the RD cells with varying concentrations of silymarin or baicalein for 1 h at 37 °C. The cell protection assays measured by the number of plaques formed indicated that silymarin exhibited only 16.6% inhibition of EV-A71 infectivity in RD cells whilst a minimal 2.2% inhibition of infectivity was observed for baicalein (Fig. 4a and b). Viral RNA copy number quantification of the supernatants collected from cell protection assay revealed that both flavonoids did not exhibit any cell protection activity against EV-A71 infection (data not shown).

### Silymarin and baicalein did not inhibit EV-A71 post infection

To determine whether silymarin and baicalein could inhibit EV-A71 post-infection, RD cells were infected with EV-A71 followed by treatment with these flavonoids. It was observed that both silymarin and baicalein were non-inhibitory when added 1 h after RD cells were infected with EV-A71 (data not shown).

### Silymarin and baicalein exhibited higher EV-A71 infectivity inhibition in combined virucidal and cell protection assays

**Fig. 5:**
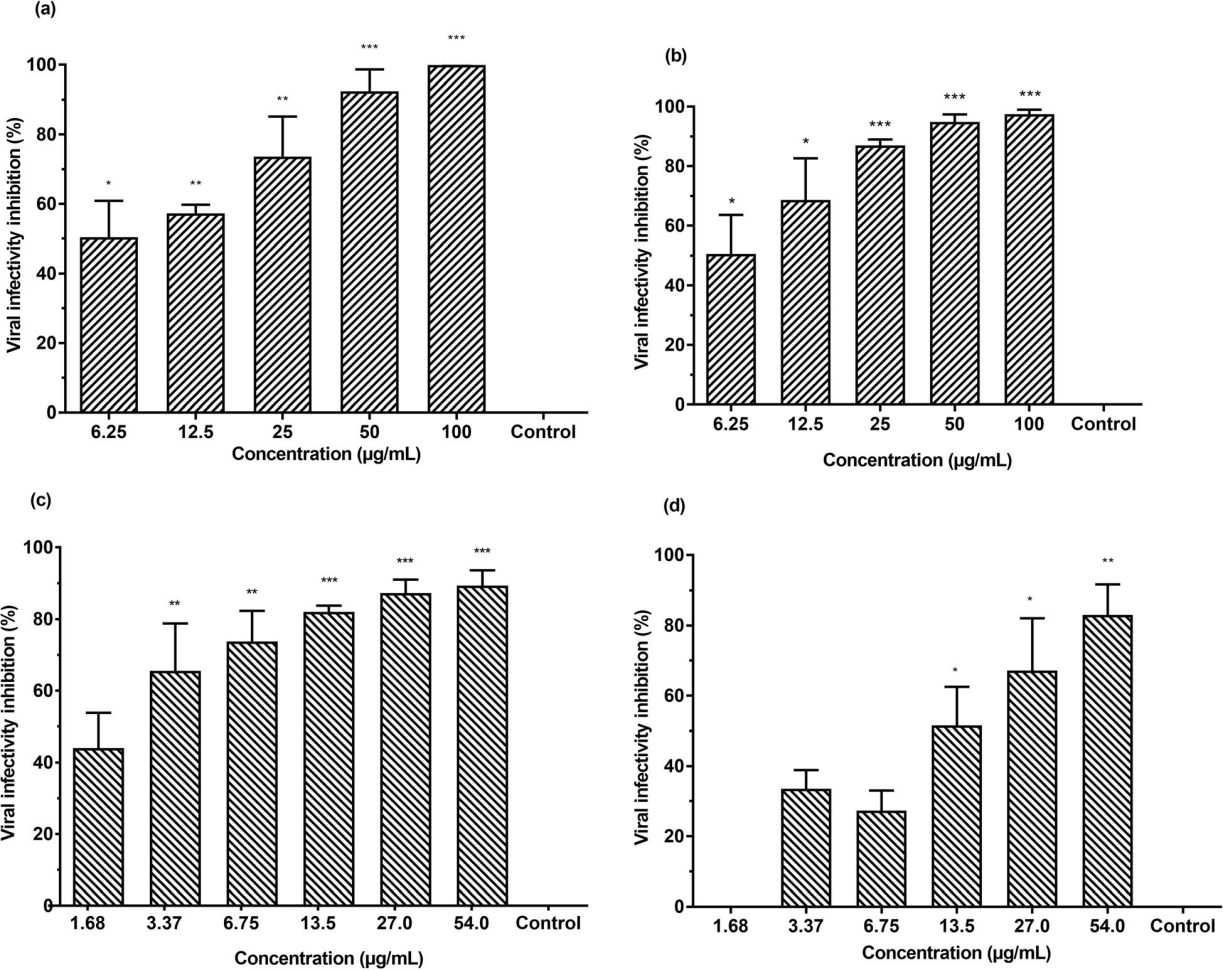
Silymarin and baicalein exhibited antiviral activities against EV-A71 in combined virucidal and cell protection assays in a dose-dependent manner. Inhibition of viral infectivity by silymarin determined by 5(**a**) plaque assays and 5(**b**) quantification of RNA copy number. Inhibition of viral infectivity by baicalein determined by 5(**c**) plaque assays and 5(**d**) quantification of RNA copy. Data presented as mean ± S.E.M. Error bars indicate the range of values obtained from three independent experiments. *p < 0.05, **p < 0.01, ***p < 0.001 indicates a significant difference compared to the negative control (0 μg/mL) analysed by *t*-test

Comprehensive assays were performed to determine the combined effects of virucidal activity and cell protection activity of silymarin and baicalein against EV-A71. In this assay, EV-A71 and RD cells were treated simultaneously with either of the flavonoids for 1 h at 37 °C. Thereafter, the flavonoid-treated RD cells were infected with the flavonoid-treated virus. The IC_50_ of silymarin was 7.99 ± 3.0 μg/mL (Table 2). The inhibition of EV-A71 infectivity was 100% when both EV-A71 and RD cells were treated with 100 μg/mL of silymarin (Fig. 5a). For baicalein, the IC_50_ was 2.27 ± 4.9 μg/mL. The highest infectivity inhibition of EV-A71 was 89.4% when both EV-A71 and RD cells were treated with 54 μg/mL of baicalein (Fig. 5c). The combined virucidal and cell protection effects of silymarin and baicalein in EV-A71 infection in RD cells were also examined by quantifying viral RNA copy number. A qRT-PCR was performed to quantify the viral RNA copy number using supernatants harvested from the comprehensive assays. Consistent with the reduction in EV-A71 plaque formations, the RNA copy number in the supernatants of EV-A71 treated with silymarin and baicalein were significantly lower than the untreated virus controls. The inhibition of EV-A71 infectivity was 97.0% when 100 μg/mL of silymarin were added (Fig. 5b) and 83.0% when 54 μg/mL of baicalein were used (Fig. 5d). The SI values of silymarin and baicalein were 20 and 185.6, respectively; indicating that these compounds are safe and efficacious for antiviral treatments (Table 2).  Inhibition of EV-A71 viral infectivity by silymarin and baicalein from virucidal, cell protection and comprehensive assays are summarized in (Table 3). Since silymarin was found to possess better virucidal activity than baicalein, we further investigated the effects of silymarin on viral attachment and viral entry.

**Table 3:** Inhibition of EV-A71 viral infectivity by silymarin and baicalein from virucidal, cell protection and comprehensive assays

Compound (Concentration)	Quantification	Virucidal assay	Cell protection assay	Comprehensive assay
Silymarin (100 μg/mL)	Plaque assay	94.0%	16.6%	100.0%
	qRT-PCR	84.3%	19.9%	97.0%
Baicalein (54 μg/mL)	Plaque assay	65.0%	2.2%	89.4%
	qRT-PCR	78.8%	0.0%	83.0%

The values presented indicated inhibitions of viral infectivity by silymarin (100 μg/mL) and baicalein (54 μg/mL) obtained from the virucidal, cell protection and comprehensive assays, respectively

### Silymarin blocked EV-A71 viral attachment to Vero cells

**Fig. 6:**
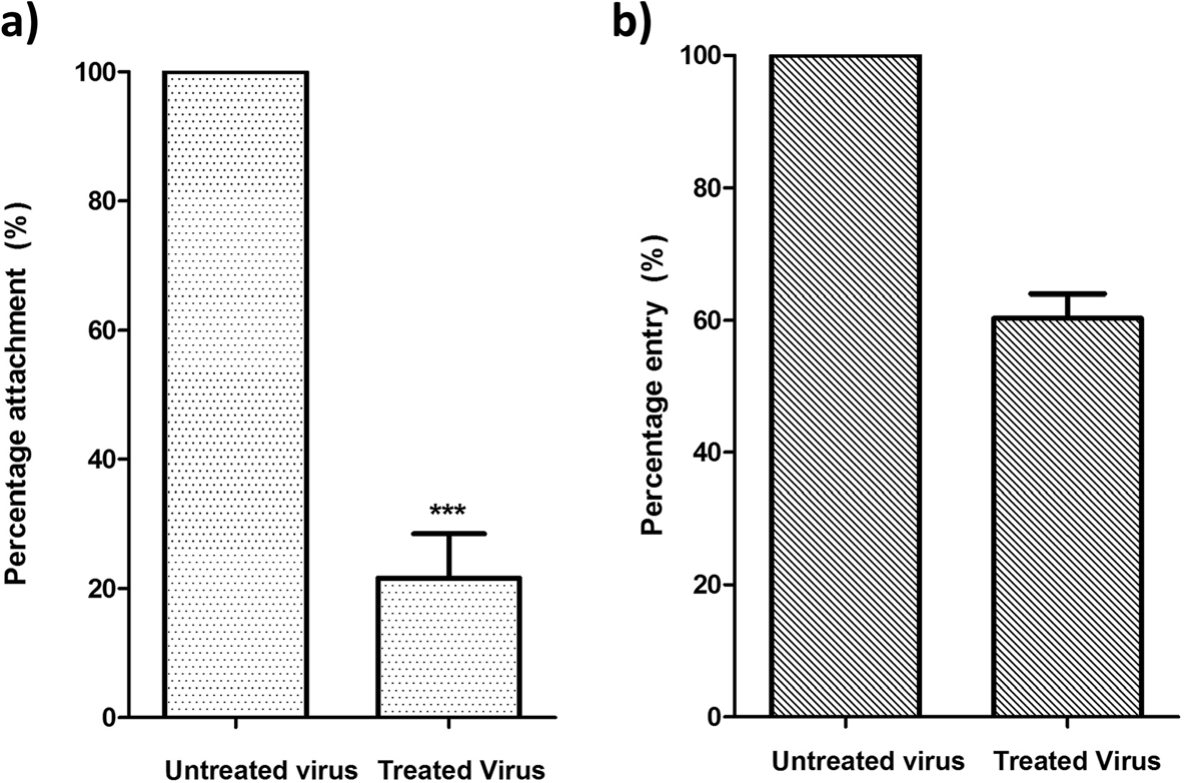
Effects of 100 μg/mL silymarin on the attachment and entry of EV-A71 to Vero cells. **a** The viral attachment inhibition was calculated as the number of plaques formed by the silymarin-treated virus over the number of plaques formed by the untreated virus. **b** Viral entry inhibition was calculated as the number of plaques formed by the virus treated with silymarin after attachment to the cells over the number of plaques formed by the untreated virus. Data presented as mean ± S.E.M. Error bars indicate the range of values obtained from three independent experiments. *** *p* < 0.001 indicates a significant difference in between groups compared by *t*-test

Since RD cells are prone to detachment when incubated at 4 °C, we performed our experiments using Vero cells that can tolerate a low-temperature environment and are susceptible to EV-A71 infection [23, 24]. To assess attachment inhibition, we co-incubated 100 μg/mL of silymarin with EV-A71 at 37 °C for 1 h and incubated the flavonoid-EV-A71 mixture or untreated EV-A71 in Vero cells at 4 °C for another hour to allow attachment of the virus to the cells. Subsequently, the inoculum was removed and the plates were incubated at 37 °C to allow the attached viruses to enter and infect the cells. Attachment assays indicated that silymarin inhibited 78% of virus attachment (Fig. 6a).

Cell cytotoxicity assay was performed to ensure that Vero cells can tolerate silymarin treatment at 100 μg/mL. It was found that silymarin did not show significant cytotoxicity up to the concentration of 200 μg/mL. The CC_50_ and CC_10_ for silymarin was 753 ± 0.17 μg/mL and 314.2 ± 0.06 μg/mL, respectively (Supplementary Figure S4, Supplementary Table ST1).

### Silymarin partially inhibited EV-A71 entry into Vero cells

For the entry assay, EV-A71 was pre-attached to the cells in the absence of silymarin at 4 °C. Silymarin was then added and the incubation temperature was shifted to 37 °C to facilitate entry of the pre-attached virus and further incubated for 1 h. The virus that did not enter the cells were inactivated using the alkaline PBS and plaques were counted after 72 h. It was observed that silymarin could partially inhibit viral entry up to 40% (Fig. 6b).

## Discussion

Despite the global distribution of HFMD caused by EV-A71, the management of children affected by this disease is primarily focused on alleviating the disease symptoms due to the absence of an effective antiviral treatment. In search for an effective antiviral agent, numerous compounds such as peptides and flavonoids have been studied for their antiviral properties against EV-A71. For example, a peptide derived from EV-A71 capsid protein VP1 designated as SP40, was shown to inhibit EV-A71 infection in various cell lines including RD, HeLa, HT-29 and Vero cells [20]. In addition, synthetically-derived flavonoids such as quercetin and nobiletin were reported to be potent inhibitors of EV-A71 in vitro [9]. Furthermore, baicalin was found to exhibit in vitro antiviral effects against the EV-A71 BrCr-TR strain in RD cells [16]. In addition to the aforementioned flavonoids, silymarin and baicalein were previously reported to have antiviral effects against other viruses. For instance, several studies have demonstrated that silymarin exhibited antiviral activities against HCV, mayaro virus and CHIKV [10, 11, 25]. Similarly, baicalein demonstrated inhibitory activities against dengue virus, Japanese encephalitis virus and influenza virus [7, 19, 26]. However, not much is known about the antiviral activities of these compounds against EV-A71. To address this, we examined the in vitro inhibitory effects of silymarin, baicalein and baicalin against EV-A71 sub-genotype B4 (strain 41) in RD cells.

In this study, the cytotoxicity of silymarin, baicalein and baicalin was evaluated. Silymarin and baicalein were found to be well tolerated by RD cells with CC_50_ values of 160.20 μg/mL and 421.46 μg/mL, respectively. Intriguingly, baicalin was found to be cytotoxic to RD cells with a low CC_50_ value of 42.17 μg/mL. This differed significantly to the CC_50_ value of 823.53 μg/mL reported in the study by Li et al. (2015) [16]. This discrepancy could be due to the use of different sources of baicalin and RD cell lines in both studies. Moreover, no antiviral effects of baicalin were observed at its non-cytotoxic concentrations (2.78 μg/mL and 5.57 μg/mL). This is most likely due to the different EV-A71 strain used in our study and the one investigated by Li et al. (2015).

Both silymarin and baicalein exhibited extracellular virucidal activity against EV-A71 with IC_50_ values of 15.20 ± 3.53 μg/mL and 30.88 ± 5.50 μg/mL, respectively. As the IC_50_ value of silymarin is lower than the IC_50_ value of baicalein, it can be considered more potent than baicalein. In addition, silymarin was able to inhibit 95% of infectivity at its highest non-toxic concentration tested (100 μg/mL) as opposed to 64% EV-A71 infectivity inhibition by baicalein at its highest non-toxic concentration tested (54 μg/mL). In the cell protection assay, silymarin displayed minimal inhibition of EV-A71 (16.6% at 100 μg/mL), while baicalein displayed no inhibition. This finding is consistent with Zhang et al. (2014) who reported that when cells were treated with 30 μM baicalein prior to EV-A71 infection, it was found to be non-effective as a prophylactic agent [27]. Both flavonoids did not inhibit EV-A71 in the post-infection assay. This finding is distinct from previous studies that indicated silymarin and baicalein mainly exerted their antiviral effects against CHIKV and HCV in post-infection assays [10, 28]. Silymarin was shown to inhibit HCV by inhibiting HCV cellular entry, viral protein expression, and infectious virus production [28]. In addition, silymarin was shown to exhibit antiviral activity against CHIKV as it was able to reduce both its replication efficiency and down-regulate the production of viral proteins involved in the replication [10]. Likewise, baicalein was able to inhibit Zika virus replication at post-infection stages [29].

In a comprehensive assay to assess the combined virucidal and cell protection effects of both flavonoids on EV-A71, we found that silymarin and baicalein exhibited higher antiviral activities against EV-A71 in comparison to the antiviral activities demonstrated in the virucidal assay or the cell protection assay alone. Further, we investigated the effective time needed for silymarin and baicalein to exert their extracellular virucidal effects on EV-A71. Our study revealed that silymarin exhibited higher extracellular antiviral activity than baicalein as silymarin required only 15 min co-incubation time to elicit more than 80% viral infectivity inhibition as opposed to baicalein that required 60 min co-incubation time to exert 60% virucidal effects against EV-A71. We also investigated the shortest time needed by silymarin to exert its virucidal effects and found that silymarin required a very minimal co-incubation time of 1 min to start exerting 65% virucidal effects on EV-A71. Consequently, our findings could suggest that antiviral activity of silymarin mainly occurred due to direct interaction of silymarin with virus surfaces to prevent viral attachment and entry. Our attachment assay indicated that the EV-A71 bound by silymarin exhibited lower infectivity in comparison to unbound EV-A71, likely due to their inability to attach to the surface of Vero cells. In the entry assay, silymarin displayed limited ability to bind to EV-A71 that were already attached to the Vero cells to detach the virus from the cell surface and prevent viral entry.

Many compounds have been shown to directly inhibit EV-A71 infectivity by targeting the structural viral proteins. These molecules designated as capsid-binding molecules inhibit viral infections either by inhibiting the viral attachment to the cellular receptors on the host cells or by stabilizing the capsid to prevent viral uncoating. For instance, pyridyl imidazolidinones, carrageenan and WIN 51711 have been shown to interact with the VP1 of EV-A71 to exert their inhibitory effects against EV-A71 [30–32]. It is possible that silymarin and baicalein employ similar mechanism and could be classified as capsid-binding molecules. From our findings, we showed that these flavonoids could specifically exert their inhibitory effects by direct binding to the EV-A71 virus, possibly through interactions with the capsid. EV-A71 capsid comprises external structural viral proteins VP1, VP2, VP3, and an internal structural viral protein VP4. The interactions between silymarin and baicalein with the EV-A71 capsid might block EV-A71 attachment to its cellular receptors such as P-selectin glycoprotein ligand-1 (PSGL-1), scavenger receptor SCARB2, heaparan sulfate, annexin2 or vimentin [33]. It is also possible that these flavonoids could block structural transitions of EV-A71, ultimately hindering EV-A71 cellular attachment and entry.

Previously, silymarin was shown to exert multiple effects against HCV by inhibiting cellular entry, viral RNA and protein expression, as well as infectious virus production [28]. In addition, silymarin was shown by Lani et al. (2015) to exhibit antiviral activity against CHIKV as it was able to reduce both CHIKV replication efficiency and down-regulate the production of viral proteins such as nsP1, nsP3 and E2E1 proteins involved in its replication [10]. In contrast to previous findings, this study revealed a novel mechanism of viral inhibition by silymarin which is through direct interactions with the virus surface to prevent viral attachment and to a lesser extent on viral entry. As opposed to silymarin, baicalein was previously shown to exert potent extracellular virucidal and prophylactic effects against dengue virus [17]. This is consistent with our current findings that it could exert extracellular virucidal effects towards EV-A71. The unique inhibitory mechanism exerted by silymarin and baicalein against EV-A71 raises the possibility of designing an effective HFMD treatment based on the combinatorial approach. For instance, this can be achieved by combining two different flavonoids to render synergistic inhibitory effects against EV-A71.

This study has implications for the overall putative mechanism of EV-A71 inhibition by flavonoids. Previously, many flavonoids were shown to inhibit EV-A71 infection by post-infection mechanisms, in particular, through the viral RNA replication machinery. Li et al. (2015) showed that baicalin reduced EV-A71 (BrCr-TR strain) infection through inhibition of EV-A71 3D polymerase expression and Fas/FasL signaling pathways [16]. Subsequently, Min et al. (2018) proposed that flavonoids derived from the flavone structures of quercetin and nobiletin affected the transcription and translation machinery of EV-A71, which is consistent with the initial suggestion that flavonoids interfered with the EV-A71 3D polymerase expression [9]. In addition, other flavonoids such as apigenin, luteolin, and 7-hydroxyisoflavone were also found to inhibit viral RNA replications at various stages of translation [27, 34]. Our study is the first to demonstrate the extracellular virucidal activities of silymarin and baicalein against EV-A71.

## Conclusion

In summary, this study revealed that natural compounds such as silymarin and baicalein potently inhibited EV-A71 infection in vitro via direct extracellular virucidal effects against EV-A71. In addition to virucidal effects, silymarin was identified to also exert its antiviral activities by significantly inhibiting EV-A71 attachment to the cells. Silymarin could have bound to the virus and interfered with its subsequent attachment to the receptor(s). Further studies to elucidate the molecular basis of extracellular virucidal effects against EV-A71 are warranted. The potential of using these two virucidal flavonoids with others in a synergistic manner should be explored, especially the combination of two or more flavonoids with different modes of inhibition mechanism against EV-A71.

## Supplementary information

**Additional file 1.** : Figure S1. Schematic representation of cytotoxic assay. Flavonoids were diluted serially in DMEM containing 2% FBS. RD or Vero cells (2 × 10^4^/well) were treated with the diluted flavonoids for 24 h. After 24 h, cytotoxicity was determined by the MTS assay using microplate reader to measure absorbance at 490 nm.

**Additional file 2 **: Figure S2. Schematic representation of antiviral assays. (**a**) **Virucidal assay**. Virus was pre-treated with flavonoid for 1 h at 37 °C. RD cells (2 × 104/well) were infected with the virus-flavonoid-mixture. The inoculum was allowed to incubate with the RD cells at 37 ° for 1 h. After 1 h, the inoculum was removed, cells were washed with PBS and maintenance media was replaced. After 24 h, the supernatant was collected. Plaque assay was performed to determine the infectious viral titers in the collected supernatant by infecting new monolayer of RD cells. RNA copy number from the supernatant was determined by qRT-PCR. (**b**) **Time course assay for virucidal activity**. Virus was pre-treated with flavonoid for different durations (1, 5, 10, 15, 20, 30, 40, 50 and 60 min) at 37 °C. RD cells (2 × 104/well) were infected with the virus-flavonoid-mixture. The inoculum was allowed to incubate with the RD cells at 37 ° for 1 h. After 1 h, the inoculum was removed, cells were washed with PBS and maintenance media was replaced. After 24 h, the supernatant was collected and infectious viral titers were quantified by plaque assay. (**c**) **Cell protection assay**. RD cells (2 × 104/well) were treated with different concentrations of flavonoid for 1 h at 37 °C. After incubation, flavonoid containing media was removed and cells were washed with PBS. Pre-treated cells were infected with EV-A71 for 1 h. The inoculum was removed, cells were washed with PBS and replaced with 2% FBS supplemented DMEM. The supernatant was collected after 24 h and the infectious viral titers were quantified by plaque assay and qRT-PCR. (**d**) **Post-infection assay**. RD cells (2 × 10^4^/well) were infected with the virus at MOI of 1 for 1 h at 37 °C. The inoculum was removed and RD cells were washed with PBS. The virus-infected cells were treated with serially diluted concentrations of flavonoid prepared in maintenance media and incubated for 24 h at 37 °C. After 24 h, the supernatant was collected and infectious viral titers were quantified by plaque assay and qRT-PCR. (**e**) **Comprehensive assay**. RD cells (2 × 10^4^/well) were pre-treated with various concentrations of flavonoid for 1 h at 37 °C. Simultaneously, the virus at MOI of 1 was pre-treated with the same concentrations of the flavonoid for 1 h at 37 °C. After incubation, flavonoid containing media was removed and the RD cells were washed with PBS. The flavonoid-treated virus was added to the pre-treated RD cells for 1 h at 37 °C. The inoculum was removed, RD cells were washed with PBS and replaced with the maintenance media (DMEM supplemented with 2% FBS). After 24 h, the supernatant was collected and infectious viral titers were quantified by plaque assay and qRT-PCR.

**Additional file 3 **: Figure S3. Schematic representation of attachment and entry assays. (**a**) **Attachment assay**. Silymarin (100 μg/mL) was pre-incubated with EV-A71 (MOI = 1) at 37 °C for 1 h. Pre-chilled Vero cells (1.5 × 105/mL) were infected with the pre-chilled silymarin-treated virus and incubated at 4 °C for 1 h to allow virus attachment. The inoculum was removed after 1 h and Vero cells were washed with PBS. CMC (1.2%, medium viscosity) overlay maintenance media was added to each well. After incubation for 3 days, the overlay media was removed. The Vero cells were washed three times with PBS, fixed with formaldehyde and stained with 0.5% crystal violet. (**b**) **Entry assay**. The virus in the absence of silymarin was added to the pre-chilled Vero cells and incubated at 4 °C for 1 h to allow virus attachment. Thereafter, the inoculum was removed after 1 h and Vero cells (1.5 × 105/mL) were washed with PBS to remove any unattached virus. Silymarin (100 μg/mL) was added to Vero cells and the temperature was shifted to 37 °C for 1 h to allow virus entry. After 1 h, the medium was removed and Vero cells were treated with alkaline PBS (pH 11) for 60 s at room temperature to inactivate the extracellular virus. After 60 s, the alkaline pH was neutralized by the addition of PBS (pH 3) in each well. Cells were then washed with serum-free media. CMC (1.2%, medium viscosity) overlay maintenance media was added to each well. After incubation for 3 days, the overlay media was removed. The Vero cells were washed three times with PBS, fixed with formaldehyde and stained with crystal violet.

**Additional file 4.** : Figure S4. Cytotoxic effects of flavonoids in Vero cells. Flavonoids (a) silymarin (b) baicalein and (c) baicalin were diluted serially in DMEM containing 2% FBS. Vero cells (2 × 104/well) were treated with the diluted flavonoid for 24 h. After 24 h, cytotoxicity was determined by the MTS assay using microplate reader to measure absorbance at 490 nm. Data are presented as mean ± S.E.M. Error bars indicate the range of values obtained from three independent experiments.

**Additional file 5.** : Supplementary Table ST1. Cytotoxicity of flavonoids against Vero cells. Different concentrations of silymarin, baicalein and baicalin were tested for cytotoxicity in Vero cells by the MTS assay. Data are presented as mean ± S.E.M.

## Data Availability

The datasets used and/or analysed during the current study are available from the corresponding author on reasonable request.

## Abbreviations

CC_10_: 10% cytotoxic concentration
CC_50_: Half maximal cytotoxic concentration;
cDNA: Complementary DNA
CHIKV: Chikungunya virus
CO_2_: Carbon dioxide
CPE: Cytopathic effect
CV-A16: Coxsackievirus 16
DMEM: Dulbecco’s Modified Eagle Medium
DMSO: Dimethyl sulfoxide
EV-A71: Enterovirus 71
HFMD: Hand, foot and mouth disease
HIV: Human immunodeficiency virus
hnRNP: Heterogeneous nuclear nucleoproteins
IC_50_: 50% inhibitory concentration
IRES: Inhibition of internal ribosome entry site
MOI: Multiplicity of infection
MTS: [3-(4,5-dimethylthiazol-2-yl)-5-(3-carboxymethoxyphenyl)-2-(4-sulfophenyl)-2H-tetrazolium]
NF-κB: Nuclear factor kappa-light-chain-enhancer of activated B cells
qRT-PCR: Quantitative real-time, reverse transcriptase polymerase chain reaction
RD: Rhabdomyosarcoma
RNA: Ribonucleic acid
S.E.M: Standard error of mean
SI: Selectivity index
TNF-α: Tissue necrotic factor α
VP: Viral protein
