# Supplementary figures and images for: Antiviral activity of silymarin in comparison with baicalein against EV-A71
Source: BMC Complement Med Ther. 2020 Mar 23;20:97. doi: 10.1186/s12906-020-2880-2 (PMC7092479; doi:10.1186/s12906-020-2880-2)

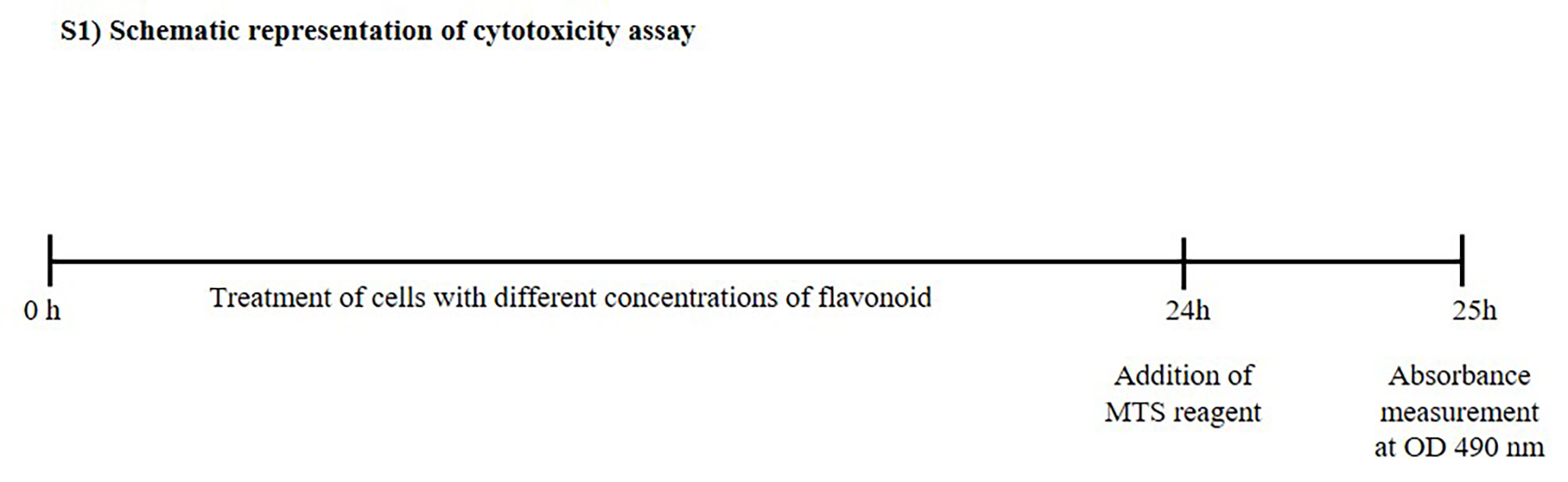

Supplement: Supplementary file 1 — Additional file 1. : Figure S1. Schematic representation of cytotoxic assay. Flavonoids were diluted serially in DMEM containing 2% FBS. RD or Vero cells (2 × 10^4^/well) were treated with the diluted flavonoids for 24 h. After 24 h, cytotoxicity was determined by the MTS assay using microplate reader to measure absorbance at 490 nm. [file 12906_2020_2880_MOESM1_ESM.tif]

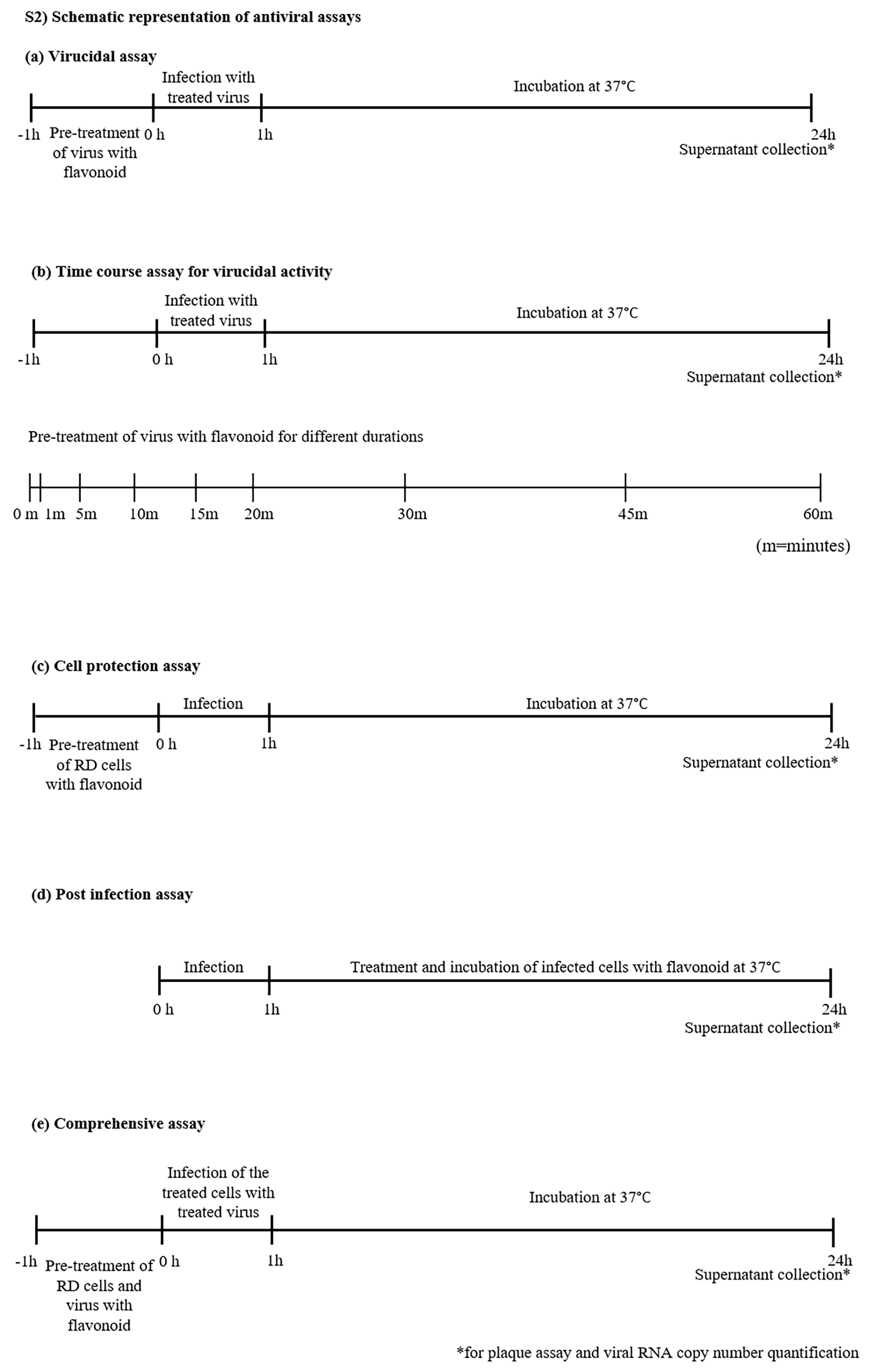

Supplement: Supplementary file 2 — Additional file 2 : Figure S2. Schematic representation of antiviral assays. (a) Virucidal assay. Virus was pre-treated with flavonoid for 1 h at 37 °C. RD cells (2 × 10^4^/well) were infected with the virus-flavonoid-mixture. The inoculum was allowed to incubate with the RD cells at 37 ° for 1 h. After 1 h, the inoculum was removed, cells were washed with PBS and maintenance media was replaced. After 24 h, the supernatant was collected. Plaque assay was performed to determine the infectious viral titers in the collected supernatant by infecting new monolayer of RD cells. RNA copy number from the supernatant was determined by qRT-PCR. (b) Time course assay for virucidal activity. Virus was pre-treated with flavonoid for different durations (1, 5, 10, 15, 20, 30, 40, 50 and 60 min) at 37 °C. RD cells (2 × 10^4^/well) were infected with the virus-flavonoid-mixture. The inoculum was allowed to incubate with the RD cells at 37 ° for 1 h. After 1 h, the inoculum was removed, cells were washed with PBS and maintenance media was replaced. After 24 h, the supernatant was collected and infectious viral titers were quantified by plaque assay. (c) Cell protection assay. RD cells (2 × 10^4^/well) were treated with different concentrations of flavonoid for 1 h at 37 °C. After incubation, flavonoid containing media was removed and cells were washed with PBS. Pre-treated cells were infected with EV-A71 for 1 h. The inoculum was removed, cells were washed with PBS and replaced with 2% FBS supplemented DMEM. The supernatant was collected after 24 h and the infectious viral titers were quantified by plaque assay and qRT-PCR. (d) Post-infection assay. RD cells (2 × 10^4^/well) were infected with the virus at MOI of 1 for 1 h at 37 °C. The inoculum was removed and RD cells were washed with PBS. The virus-infected cells were treated with serially diluted concentrations of flavonoid prepared in maintenance media and incubated for 24 h at 37 °C. After 24 h, the supernatant was collected and infec [file 12906_2020_2880_MOESM2_ESM.tif]

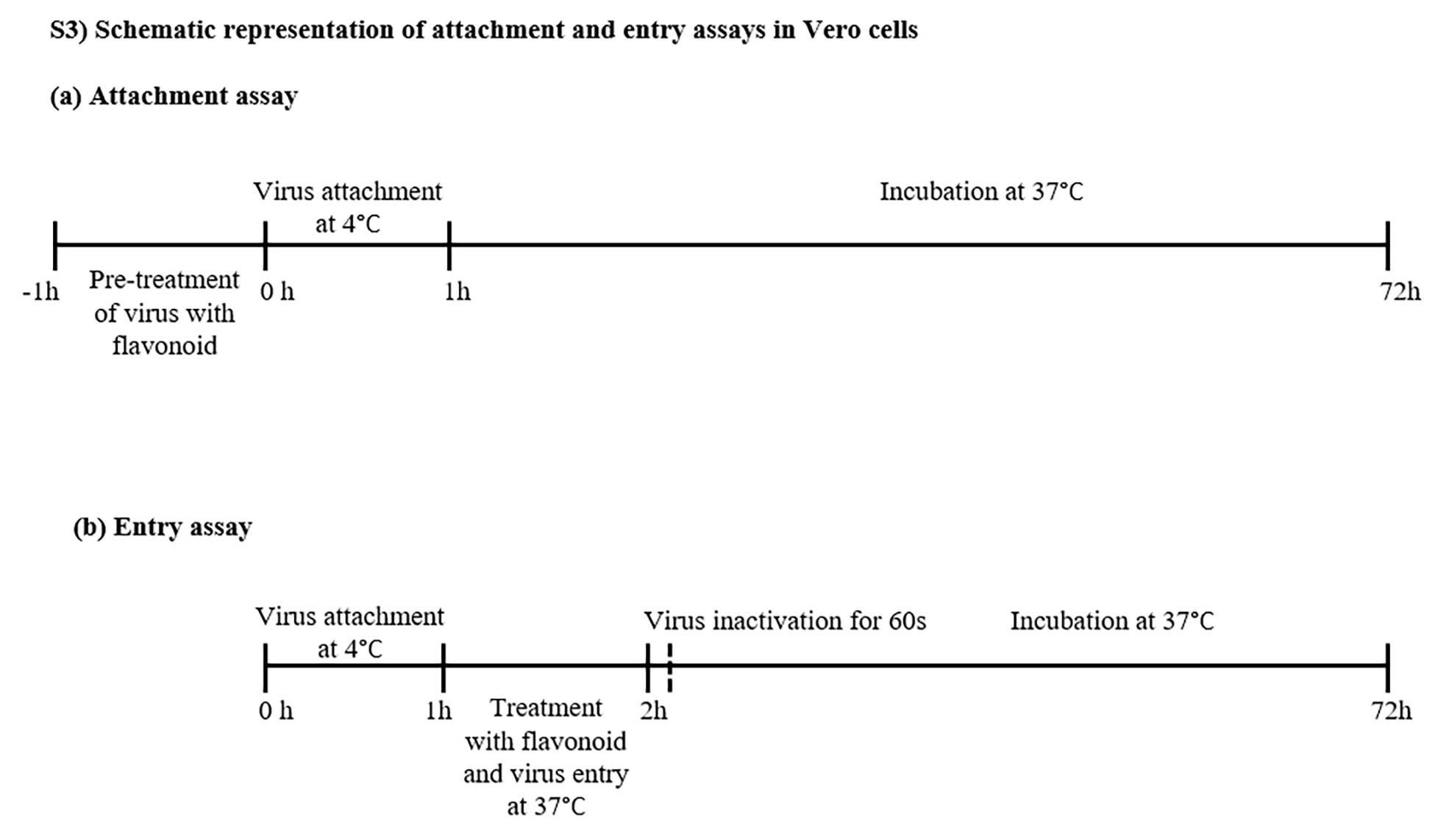

Supplement: Supplementary file 3 — Additional file 3 : Figure S3. Schematic representation of attachment and entry assays. (a) Attachment assay. Silymarin (100 μg/mL) was pre-incubated with EV-A71 (MOI = 1) at 37 °C for 1 h. Pre-chilled Vero cells (1.5 × 10^5^/mL) were infected with the pre-chilled silymarin-treated virus and incubated at 4 °C for 1 h to allow virus attachment. The inoculum was removed after 1 h and Vero cells were washed with PBS. CMC (1.2%, medium viscosity) overlay maintenance media was added to each well. After incubation for 3 days, the overlay media was removed. The Vero cells were washed three times with PBS, fixed with formaldehyde and stained with 0.5% crystal violet. (b) Entry assay. The virus in the absence of silymarin was added to the pre-chilled Vero cells and incubated at 4 °C for 1 h to allow virus attachment. Thereafter, the inoculum was removed after 1 h and Vero cells (1.5 × 10^5^/mL) were washed with PBS to remove any unattached virus. Silymarin (100 μg/mL) was added to Vero cells and the temperature was shifted to 37 °C for 1 h to allow virus entry. After 1 h, the medium was removed and Vero cells were treated with alkaline PBS (pH 11) for 60 s at room temperature to inactivate the extracellular virus. After 60 s, the alkaline pH was neutralized by the addition of PBS (pH 3) in each well. Cells were then washed with serum-free media. CMC (1.2%, medium viscosity) overlay maintenance media was added to each well. After incubation for 3 days, the overlay media was removed. The Vero cells were washed three times with PBS, fixed with formaldehyde and stained with crystal violet. [file 12906_2020_2880_MOESM3_ESM.tif]

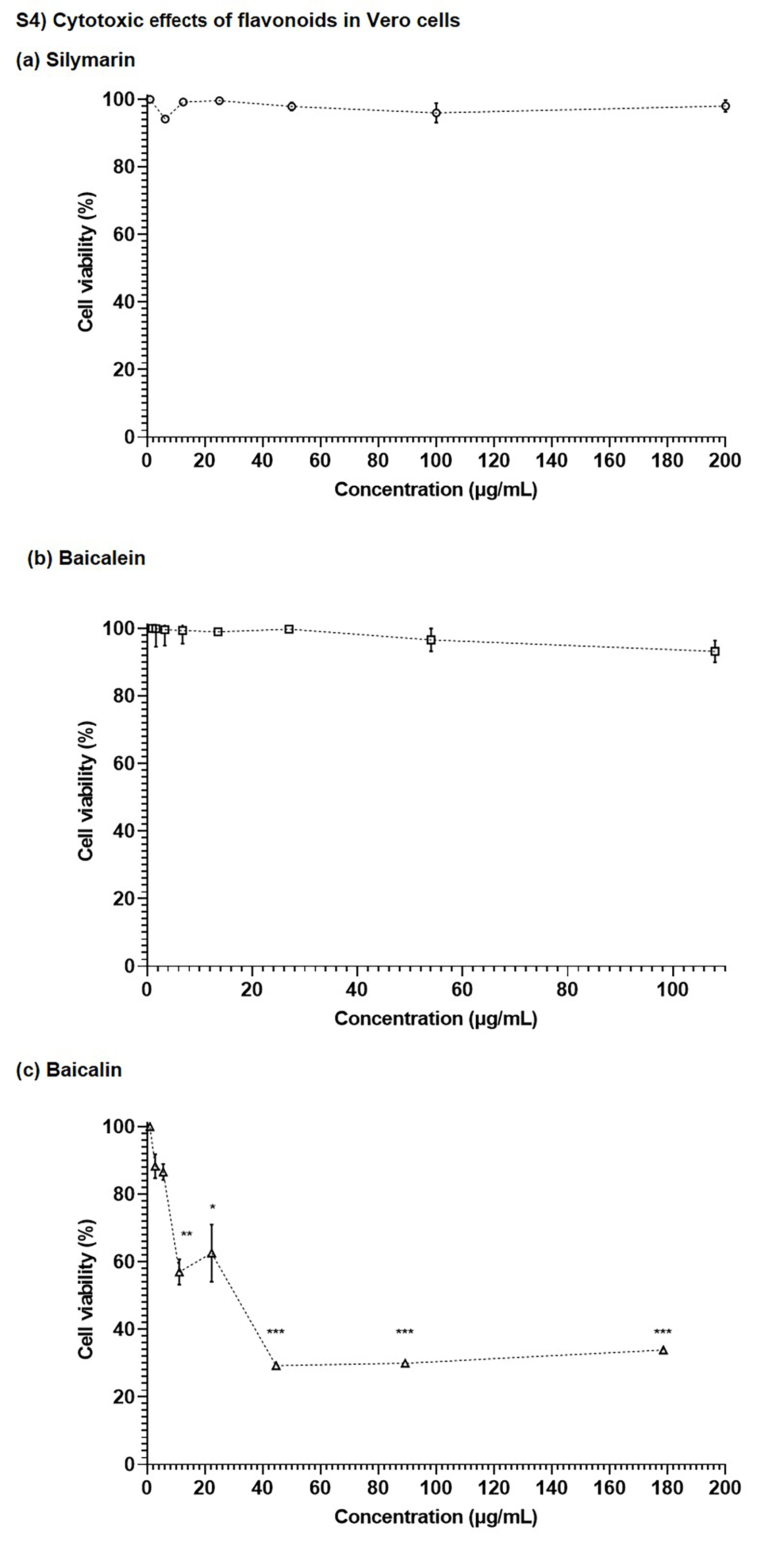

Supplement: Supplementary file 4 — Additional file 4. : Figure S4. Cytotoxic effects of flavonoids in Vero cells. Flavonoids (a) silymarin (b) baicalein and (c) baicalin were diluted serially in DMEM containing 2% FBS. Vero cells (2 × 10^4^/well) were treated with the diluted flavonoid for 24 h. After 24 h, cytotoxicity was determined by the MTS assay using microplate reader to measure absorbance at 490 nm. Data are presented as mean ± S.E.M. Error bars indicate the range of values obtained from three independent experiments. [file 12906_2020_2880_MOESM4_ESM.tif]
