# Supplementary material for: Antiviral activity of silymarin in comparison with baicalein against EV-A71
Source: BMC Complement Med Ther. 2020 Mar 23;20:97. doi: 10.1186/s12906-020-2880-2 (PMC7092479; doi:10.1186/s12906-020-2880-2)
Supplement: Supplementary file 5 — Additional file 5. : Supplementary Table ST1. Cytotoxicity of flavonoids against Vero cells. Different concentrations of silymarin, baicalein and baicalin were tested for cytotoxicity in Vero cells by the MTS assay. Data are presented as mean ± S.E.M. [file 12906_2020_2880_MOESM5_ESM.docx]

**Supplementary Table ST1**. Cytotoxicity of flavonoids against Vero cells.

| Compound | CC_50_ µg/mL | CC_10_ µg/mL |
| --- | --- | --- |
| Silymarin | 753 ± 0.17 | 314.2 ± 0.06 |
| Baicalein | 828.7 ± 0.93 | 148.9 ± 0.25 |
| Baicalin | 30.47 ± 0.06 | 1.718 ± 0.16 |

Different concentrations of silymarin, baicalein and baicalin were tested for cytotoxicity in Vero cells by the MTS assay. CC_50_ refers to as half maximal cytotoxic concentration whereas CC_10_ is the 10% cytotoxic concentration to the cells. Data are presented as mean ± S.E.M.
